# Global prevalence of myasthenia gravis and the effectiveness of common drugs in its treatment: a systematic review and meta-analysis

**DOI:** 10.1186/s12967-021-03185-7

**Published:** 2021-12-20

**Authors:** Nader Salari, Behnaz Fatahi, Yalda Bartina, Mohsen Kazeminia, Reza Fatahian, Payam Mohammadi, Shamarina Shohaimi, Masoud Mohammadi

**Affiliations:** 1 Department of Biostatistics, School of Health, Kermanshah University of Medical Sciences, Kermanshah, Iran; 2 Student Research Committee, Kermanshah University of Medical Sciences, Kermanshah, Iran; 3 Department of Translation Studies, Faculty of Literature, Istanbul University, Istanbul, Turkey; 4 Department of Neurosurgery, School of Medicine, Kermanshah University of Medical Sciences, Kermanshah, Iran; 5 Department of Neurology, School of Medicine, Kermanshah University of Medical Sciences, Kermanshah, Iran; 6 Department of Biology, Faculty of Science, University Putra Malaysia, Serdang, Selangor Malaysia; 7 Cellular and Molecular Research Center, Gerash University of Medical Sciences, Gerash, Iran

**Keywords:** Myasthenia gravis, MS, Prevalence, Drug, Systematic review, Meta-analysis

## Abstract

**Background:**

Myasthenia gravis is a neuromuscular autoimmune disorder characterized by weakness and disability in the voluntary muscles. There have been several preliminary studies on the epidemiology of myasthenia gravis in different parts of the world and the effectiveness of common drugs in its treatment, but there has been no comprehensive study of the efficacy of common drugs in the treatment of myasthenia gravis. Therefore, this study aimed to determine the epidemiology of myasthenia gravis globally and the effectiveness of common drugs in its treatment using systematic review and meta-analysis.

**Methods:**

Research studies were extracted from IranDoc, MagIran, IranMedex, SID, ScienceDirect, Web of Sciences (WoS), ProQuest, Medline (PubMed), Scopus and Google Scholar based on Cochran's seven-step guidelines using existing keywords extracted in MeSH browser. The I^2^ test was used to calculate the heterogeneity of studies, and Begg and Mazumdar rank correlation tests were used to assess publication bias. Data were analyzed using Comprehensive Meta-Analysis software (Version 2).

**Results:**

In the search for descriptive studies based on the research question, 7374 articles were found. After deleting articles unrelated to the research question, finally, 63 articles with a sample size of 1,206,961,907 people were included in the meta-analysis. The prevalence of MG worldwide was estimated to be 12.4 people (95% CI 10.6–14.5) per 100,000 population. For analytical studies on the effectiveness of common myasthenia gravis drugs, 4672 articles were found initially, and after removing articles unrelated to the research question, finally, 20 articles with a sample size of 643 people in the drug group and 619 people in the placebo group were included in the study. As a result of the combination of studies, the difference between the mean QMGS score index after taking Mycophenolate and Immunoglobulin or plasma exchange drugs in the group of patients showed a significant decrease of 1.4 ± 0.77 and 0.62 ± 0.28, respectively (P < 0.01).

**Conclusion:**

The results of systematic review of drug evaluation in patients with myasthenia gravis showed that Mycophenolate and Immunoglobulin or plasma exchange drugs have positive effects in the treatment of MG. It also represents the positive effect of immunoglobulin or plasma exchange on reducing SFEMG index and QMGS index and the positive effect of Mycophenolate in reducing MG-ADL index, SFEMG and Anti-AChR antibodies index. In addition, based on a meta-analysis of the random-effect model, the overall prevalence of MG in the world is 12.4 people per 100,000 population, which indicates the urgent need for attention to this disease for prevention and treatment.

## Background

Myasthenia gravis (MG) is a neuromuscular disease characterized by weakness in the voluntary muscles [1, 2]. This disease has different symptoms that vary in different patients depending on the degree of involvement of the striated muscles. The most common type of symptom in patients with myasthenia gravis is ocular symptoms, which appear as ptosis and diplopia. These symptoms usually occur at the end of the day and follow activities such as watching TV or driving is more common, and excessive fatigue has been reported due to frequent activity in patients with this disease [3].

Myasthenia gravis is an autoimmune disease that connects the nerve to the muscle (NMJ) [4], which is produced by different antibodies against synaptic membrane proteins [5]. This is usually more than 85% of cases and is caused by a type of antibody against the skeletal muscle acetylcholine receptor (AChR-Ab) [6, 7]. However, components other than AChR, such as muscle-specific tyrosine kinase receptor (MuSK) or lipoprotein-associated protein 4 (LRP4), may also be targeted for the autoimmune attack [6, 8, 9].

Based on the mechanism of autoimmune disease and antibodies, invasive skeletal muscle molecules, thymus status, genetic characteristics, disease phenotype and response to treatment, myasthenia gravis is divided into early and late ocular subtypes (OMG), seronegative, thymoma, LRP4, MuSk. Diagnosis of MG subtype influences treatment decisions and disease prognosis [10, 11]. Approximately 50% of patients with OMG develop general myasthenia gravis (GMG) over a 2-year period, which affects other muscles and manifests as weakness and ocular symptoms [12].

According to a systematic population-based study, CAR et al. [13] estimated the incidence and prevalence of MG at 54 per million and 77.7 per million, respectively. However, significant changes have been reported in various studies. The incidence of this disease has shown a range between 1.77 and 21.3 per million people and the prevalence of 15 to 179 million people [13]. A large number of epidemiological studies, mainly in Western Europe and Asia, reported significant differences in the incidence and prevalence of MG. The incidence of myasthenia gravis ranged from 1.7 to 30 per million per year [14–17].

The disease has two age peaks: age 40–40 years, which mainly affects women, and another 80–60 years, which occurs equally in men and women [4].

Current treatment options mainly include acetylcholinesterase inhibitors, glucocorticoids (GC), intravenous immunoglobulin (IVIg), plasma replacement (PLEX), thymectomy, and immunosuppressive agents including azathioprine prednisone, cyclosporine, cyclosporine, and cyclosporine [18–24]. However, the use of corticosteroids and immunosuppressants such as azathioprine manages MG. However, many patients do not tolerate or respond adequately to these drugs, and long-term treatment with GC is associated with a significant risk of side effects such as diabetes, obesity, and high blood pressure. This has led to the introduction of newer immunosuppressants such as mycophenolate mofetil (MMF) [12, 25–27].

About 15% of patients with general myasthenia gravis do not respond to immunosuppressants and require intravenous immunoglobulin (IVIg) or plasma replacement (PLEX) to improve their symptoms [28, 29]. Intensified cases and myasthenia gravis crisis also require immediate treatment due to poor swallowing or respiratory failure that threatens the lives of these patients and muscle defects that may be a major disability for their daily activities [30].

Therefore, additional immunosuppression is often treated with Plasma Freesia (PLEX) and intravenous immunoglobulin (IVIg) to relieve symptoms. Plasma freeze uses filtration to kill pathological antibodies used in patients with myasthenia gravis and severe MG [31–33].

Myasthenia gravis has high direct health care costs (including long-term treatment and periodic hospitalization costs) and indirect costs such as loss of income and reduced care productivity [34]. Therefore, accurate identification of patients with MG is vital for organizing health care services and implementing preventive health measures. Many early articles have been done on the prevalence of myasthenia gravis and the effect of different drugs on the treatment process, but appropriate policy to control, diagnose and treat this disease requires coherent, accurate and uniform information. Therefore, the present study was performed to estimate the prevalence of myasthenia gravis globally and determine the effectiveness of the most common drugs in the treatment of patients by systematic review and meta-analysis.

## Methods

The present systematic review and meta-analysis were conducted according to the Cochrane seven-step approach, including selecting research questions, determining inclusion and exclusion criteria, identifying descriptive articles, selecting studies, qualitative evaluation of studies, data extraction, analysis and interpretation of findings [35].

### Research question and determining the keywords of the descriptive section

According to the research question in the descriptive section, "How has the prevalence of MG in the world changed?" The population included: MG patients, Outcome included MG prevalence, Time or duration Included: Date of publication of the first related article until 15 November 2020 and type of study (study design) Included: cross-sectional (descriptive) studies. Keywords were extracted from the MeSH browser. Keywords related to the studied population (P): Myasthenia Gravis, MG and outcome keywords (O); Prevalence and Epidemiology.

### Research question and determining the keywords of the analytical section

According to the research question of the analytical section, "What is the effectiveness of Corticosteroids, Mycophenolate and Immunoglobulin or plasma exchange drugs in the treatment of MG?" According to the PICO guidelines, the study population (Population) includes patients with MG, intervention (intervention) including Corticosteroids, Mycophenolate and Immunoglobulin or plasma exchange, analogous (Comparison) including QMGS index score, Anti-AChR antibodies, SFEMG And MG-ADL before and after the intervention, the outcome (Outcome) included: changes in QMGS, Anti-AChR antibodies, SFEMG and MG-ADL after the intervention. Keywords were extracted from the MeSH browser according to PICO instructions. Keywords related to the study population (P): Myasthenia Gravis, MG Keywords related to the intervention (I); Corticosteroids, Corticotropin, Alternate-day prednisone, Methylprednisone, Prednisolone, Mycophenolate, Immunoglobulin or plasma exchange, Intravenous immunoglobulin, IVIG and keywords related to analogy (C) and outcome (O); QMGS, QMG, Anti-AChRantibodies, Anti-AChR ab, SFEMG and MG-ADL.

### Criteria for inclusion and exclusion according to the descriptive research question

Cross-sectional (descriptive) studies in chronic patients reporting the prevalence of MG in different parts of the world published in English, and the full text was available. Observational studies, cohort, case–control, analytical and interventional studies, case reports, short reports, letters to the editor and studies unrelated to the research question were excluded from the study.

### Criteria for inclusion and exclusion according to the research question of the analytical section

Clinical trial studies that reported the mean and standard deviation of the effect of Corticosteroids, Mycophenolate and Immunoglobulin or plasma exchange on at least one of the indicators of QMGS, Anti-AChR antibodies, SFEMG and MG-ADL in patients with MG, in Persian and were printed in English and their full text was available and included in the study. Descriptive studies, cross-sectional studies, reviews, case reports, short reports, letters to the editor, and other studies unrelated to the research question were excluded from the study.

### Articles identification

To find studies related to research questions, four Persian databases, including IranDoc, MagIran, IranMedex and SID and five international databases: ScienceDirect, Web of Science (WoS), ProQuest, Medline (PubMed), Scopus were searched. The Google Scholar scientific search engine was reviewed for final review. No time limit was set for the search to retrieve the relevant research, so all articles published by November 15, 2020, were reviewed. The search was limited to studies published in Persian and English. The search strategy in each database was determined using Advanced Search (Advanced Search) with the help of all possible keyword combinations with the help of (AND) and (OR) operators. For example, the search strategy in the PubMed database for the descriptive part of the research was determined as follows:

(((Prevalence [Title/Abstract]) OR (Epidemiology [Title/Abstract])) AND (Myasthenia Gravis [Title/Abstract])) OR (chronic patients [Title/Abstract]) OR (MG [Title/Abstract]).

Also, the search strategy in the PubMed database for the analytical part of the research was determined as follows:

((((((((((((((((Corticosteroids[Title/Abstract]) OR (Corticotropin[Title/Abstract])) OR (Alternate-day prednisone[Title/Abstract])) OR (Methylprednisone [Title/Abstract])) OR (chronic patients [Title/Abstract]) OR (Prednisolone[Title/Abstract])) OR (Mycophenolate[Title/Abstract])) OR (Immunoglobulin[Title/Abstract] OR plasma exchange[Title/Abstract])) OR (Intravenous immunoglobulin[Title/Abstract])) OR (IVIG[Title/Abstract])) AND (QMGS[Title/Abstract])) OR (QMG[Title/Abstract])) OR (Anti-AChR antibodies[Title/Abstract])) OR (Anti-AChR ab[Title/Abstract])) OR (SFEMG[Title/Abstract])) OR (MG-ADL[Title/Abstract])) AND (Myasthenia Gravis[Title/Abstract])) OR (MG[Title/Abstract]).

In order to access the latest published studies, an alert was created on several databases, including PubMed and Scopus, to check if new articles were published during the study. Also, in order to access all related studies, the sources of articles that met the inclusion criteria were manually reviewed. To avoid errors, all steps of article search, study selection, qualitative evaluation and data extraction were performed independently by two researchers (M.K. and B.F.). If there was a difference of opinion between the researchers regarding the inclusion of the article in the study, in order to avoid the risk of bias for specific studies, first a final agreement was reached through discussion and in some cases with the participation and opinion of a third party (MM).

### Selection of studies based on entry and exit criteria

Based on the 4-step PRISMA process, including article identification, screening, eligibility, and inclusion in the study, three researchers reviewed this process as follows, and studies were selected based on inclusion and exclusion criteria. All articles found in each database were transferred to EndNote X8 software. After completing the search in all the databases, the articles repeated in different databases were deleted. Then, in order to avoid the risk of bias in selecting studies, the names of the authors and the titles of the journals were removed, and a checklist was prepared based on the titles and abstracts of the studies. In the next step, two authors (M.K. and B.F.) independently examined the title and abstract of the studies and eliminated studies that were not related to the research based on the inclusion and exclusion criteria of the study and in case of discrepancy, it was examined by the third researcher (M.M). Studies whose full text was not found were also excluded from the systematic review and meta-analysis process. The full text of all remaining articles was then evaluated. Studies that did not meet the inclusion criteria based on the research question were excluded.

### Qualitative evaluation of descriptive studies

Qualitative evaluation of studies was performed using the STROBE checklist, a suitable tool for the qualitative assessment of descriptive studies. This checklist has 22 general items, each of which has sub-items (32 sub-items in total) and to evaluate different parts of a study, including title and abstract, study objectives, problem statement, study type, sampling method, study statistical population, the sample size is the definition of variables, tools for collecting study data, statistical analysis, findings and discussion. In order to rate the articles, if each article referred to the items considered in the checklist, it was given a score of 1, and if it was not mentioned, a score of zero was given. The minimum and maximum scores in this checklist are 0 and 32, respectively. Articles with scores of 16 and above were considered high and medium quality studies and were included in the systematic review and meta-analysis process, and articles with scores below 16 were considered low-quality studies [36].

### Qualitative evaluation of analytical studies

Qualitative evaluation of studies was performed using the CONSORT checklist, a suitable tool for the qualitative assessment of interventional studies. This checklist has 25 general items, each with minor items (a total of 37 minor items). Different sections include Title and Abstract, Introduction and Background, Methods, Participants, Interventions, Objectives, Consequences, Sample Size, Randomization, How to Assign Participants, Blind Allocation, Execution, Blindness of Study, Statistical Methods, Results, Flow Participants' presence, sampling method, initial data of the number of people analyzed, consequences and estimates, auxiliary analysis, adverse reactions, explanations, interpretation, generalizability and general evidence. In this study, all general checklist items were reviewed by two authors (M.K. and B.F.). In order to rate the articles, if each article referred to the items in the checklist, it was given a score of 1, and if it was not mentioned, a score of zero was given. The minimum and maximum scores in this checklist are 0 and 37, respectively. Studies with 75% or more of the maximum achievable score (score greater than or equal to 27) with “high quality”, studies with a score between 75 and 50% (score 18–26) as “average quality” and studies with a score below 50% (less than or equal to 17) were considered “low quality” studies [37]. Based on this checklist, medium and high-quality articles were included in the study, and low-quality articles were excluded.

### Data extraction

After selecting the studies to enter the systematic review and meta-analysis process, the data were extracted, and the studies were summarized. For this purpose, two electronic checklists (one for the descriptive section and one for the analytical section) were prepared. The various items in the descriptive checklist included: name of the first author, year of publication and year of the report, place of study, age, sample size and prevalence, and various items in the analytical checklist, including the name of the first author, year of publication, place of research, the sample size of the drug group and the placebo group was the type of drug, mean and standard deviation before and after the intervention.

### Statistical analysis of the descriptive part

To analyze and combine the results of different studies, in each study, the prevalence of MG was considered as the probability of binomial distribution and its variance was calculated through binomial distribution. Heterogeneity of studies was assessed using the I^2^ test, and the random-effects model was used in the case of the I^2^ index above 50%. In this model, parametric changes between studies are also considered in the calculations, so it can be said that the results of this model in heterogeneous conditions can be more generalized than the model with a fixed effect. Publication bias assessment was performed using Funnel Plot and Begg and Mazumdar rank correlation test. Data were analyzed using Comprehensive Meta-Analysis (Version 2) software, and the significance level of the test was P < 0.05.

### Statistical analysis of the analytical section

In this study, the standardized mean difference was calculated. The main outcome of this study was the mean score of the studied indicators before and after the intervention in patients with MG. As a result, the mean and standard deviation of the studied indices before and after the intervention were extracted. I^2^ index was used to evaluate heterogeneity. Funnel Plot and Begg and Mazumdar rank correlation tests were used to assess the publication bias. The significance level of the test was considered 0.1. Data were analyzed using Comprehensive Meta-Analysis software (Version 2).

## Results

### Descriptive part of the study

**Fig. 1:**
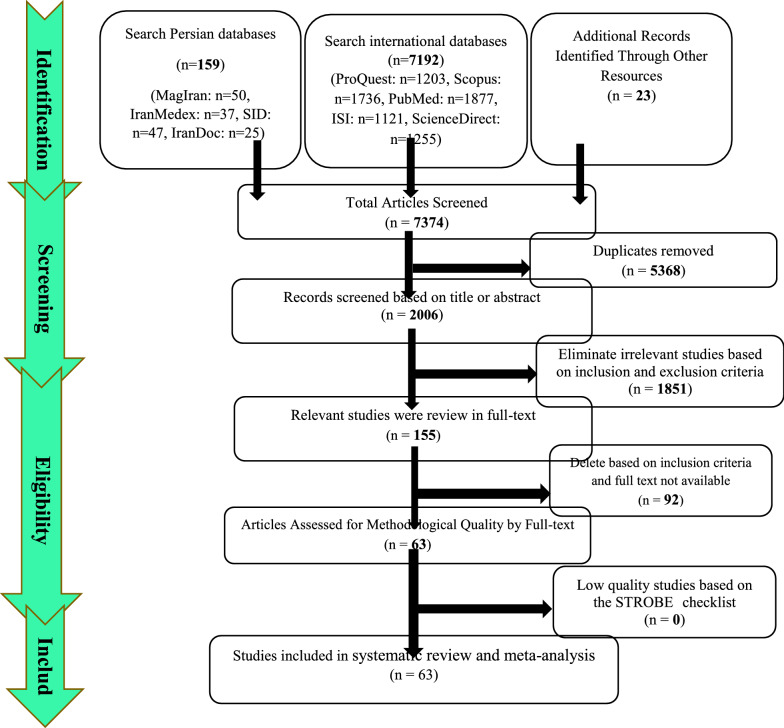
Preferred Reporting Items for Systematic Reviews and Meta-Analyses (PRISMA 2009) flow diagram Descriptive section

Summary of how articles enter meta-analysis: In the first stage, 7374 articles (7192 articles in international databases, 159 articles in Persian databases and 23 studies in reviewing article sources) were found, of which 5368 studies were repeated in different databases were removed. A total of 2006 studies were entered the in the screening stage and 1851 articles were deleted based on the inclusion and exclusion criteria by reviewing the title and abstract of the study. In the next stage (competency assessment), out of the remaining 155 studies from the screening stage, 92 articles were removed by reviewing the full text of the article because it was not relevant to the research. The quality evaluation of 63 articles included in this study was performed using the STROBE checklist, all of which were of medium and high quality according to the criteria of this tool. Thus, 63 articles related to the descriptive part of the study entered the process of systematic review and meta-analysis (Fig. 1).

#### General characteristics of the studies

The total sample size of the studies was 1,206,961,907 people. The studies were published between 1969 and November 15, 2020. There were 8 studies in Asia, 42 in Europe, 7 in the United States, 5 in Africa, and 1 in Australia. Summary of study characteristics, including the name of the first author, year of publication and year of the report, place of study, mean age of patients, sample size and prevalence of MG, are reported in Table 1.

**Table 1:** General characteristics of descriptive studies

First Author, year, Reference	Report year	Country	Age (years)	Sample size	Prevalence per hundred thousand people
Murai-1, 2011, [38]	1987	Japan	–	117,647,059	5.09
Murai-2, 2011, [38]	2006	Japan	–	127,966,102	11.79
Nemet-1, 2014, [39]	2014	Israel	–	475,097	46.93
Nemet-2, 2014, [39]	2014	Israel	–	52,160	47.92
Park-1, 2016, [40]	2010	Korea	–	50,744,570	9.67
Park-2, 2016, [40]	2011	Korea	–	50,750,469	10.66
Lee-1, 2016, [41]	2010	Korea	–	47,990,761	10.42
Lee-2, 2016, [41]	2011	Korea	–	49,779,440	11.03
Lee-3, 2016, [41]	2012	Korea	–	50,004,441	12.4
Lee-4, 2016, [41]	2013	Korea	–	50,219,669	12.96
Lee-5, 2016, [41]	2014	Korea	–	50,423,955	12.99
Okinaka, 1969, [42]	1966	Japan	–	838,000	1.78
Araki, 1983, [43]	1982	Japan	–	537,313	6.7
Kondo, 1988, [44]	1985	Pakistan	–	1,038,462	2.59
Yu, 1992, [45]	1987	China	–	4,860,000	5.34
Zieda, 2018, [46]	2018	UK	46	1986 096	11.37
Lavrnic-1, 2013, [47]	2013	Serbia	47.9 ± 19.8	1,338,161	31.76
Tola, 1989, [48]	1989	Italy	47.8 ± 18.1	370,374	10.52
Montomoli, 2012, [49]	2012	Italy	58.3 ± 17.0	495,833	24
Cetin, 2012, [50]	2012	Austria	–	8,363,040	7.99
Storm-1, 1984, [51]	1951	Norway	–	3,100,000	2
Storm-2, 1984, [51]	1961	Norway	–	3,463,415	4.09
Storm-3, 1984, [51]	1971	Norway	–	3,794,872	9.98
Storm-4, 1984, [51]	1981	Norway	–	4,107,063	8.98
Westerberg, 2020, [52]	2020	Sweden	60	13,119,113	36.1
Kalb, 2002, [53]	2002	Sweden		1,783,428	14.07
Aiello, 1997, [54]	1997	Italy	44 ± 16.6	268,926	11.15
Guidetti, 1998, [55]	1998	Italy	50.5 ± 19.8	427,493	10.29
Foldvari, 2015, [56]	2015	Hungary	60	8,259,048	2.76
Zivadinov, 1998, [57]	1998	Italy	67.7–139.6	323,232	9.9
Bettini, 2017, [58]	2017	Argentina	63.3 ± 20	978,313.561	0.006
Andersen, 2014, [59]	2014	Norway	–	4,725,190	13.1
Aragonès, 2017, [60]	2017	Spain	–	155,062	32.89
Salvado, 2016, [61]	2016	Spain	59.44 ± 29.35	462	3463
Christensen-1, 1993, [62]	1993	Denmark	–	280,000,000	7.8
Robertson, 1998, [63]	1998	England	–	684,000	14.6
Garland, 1956, [64]	1955	UK	–	500,000	3.6
Pennington, 1961, [65]	1958	UK	–	1,500,000	2.13
Gudmundsson, 1968, [66]	1963	Iceland	–	187,000	6.41
Oosterhuis, 1977, [67]	1965	Holland	–	860,000	5.58
Hokkanen, 1969, [68]	1968	Finland	–	4,493,392	2.55
Giagheddu, 1989, [69]	1986	Italy	–	2,444,444	4.5
D'Alessamdro-1, 1991, [70]	1987	Italy	–	914,463	7.21
D'Alessamdro-2, 1991, [70]	1988	Italy	–	370,374	10.52
Sorensen, 1989, [71]	1987	Denmark	–	230,760	12.56
Somnier-1, 1991, [72]	1988	Eastern Denmark	–	2,298,701	17.66
Christensen-2, 1998, [73]	1990	Western Denmark	–	2,800,000	7.85
Ferrari, 1992, [74]	1990	Italy	–	446,914	8.27
Krivopusk, 1991, [75]	1991	Russia	–	655,738	3.5
Lavrnic-2, 1999, [76]	1992	Serbia	–	1,530,864	7.64
Kyriallis, 1995, [77]	1994	Cyprus	–	600,000	17.5
Holtsema, 2000, [78]	1995	Netherlands Antilles	–	229,800	6.52
Villagr, 1997, [79]	1996	Spain	–	81,507	8.58
Oopik, 2003, [80]	1997	Estonia	–	1,462,130	14.22
Wirtz, 2003, [81]	2000	Southern Holland	–	1,725,317	10.95
Kotov-1, 2006, [82]	2001	Southern Holland	–	12,000,000	8.96
Kotov-2, 2006, [82]	2004	Southern Holland	–	1,778,564	14.22
Eaton, 2007, [17]	2001	Denmark	–	5,472,032	17.85
Somnier-2, 2005, [83]	1999	Eastern Denmark	–	2,298,701	16.35
Poulas, 2001, [84]	1997	Greece	–	10,475,873	7.06
Niks-1, 2007, [85]	2004	Netherlands	–	1,778,564	8.99
Niks-2, 2007, [85]	2004	Netherlands	–	1,778,564	0.28
Tsiamalos, 2009, [86]	2006	Greece	–	11,293,282	0.29
Breiner-1, 2016, [15]	1996	Canada	–	8,586,605	16.56
Breiner-2, 2016, [15]	1997	Canada	–	8,734,231	17.79
Breiner-3, 2016, [15]	1998	Canada	–	8,884,264	18.79
Breiner-4, 2016, [15]	1999	Canada	–	9,055,208	19.86
Breiner-5, 2016, [15]	2000	Canada	–	9,247,809	20.79
Breiner-6, 2016, [15]	2001	Canada	–	9,453,075	21.59
Breiner-7, 2016, [15]	2002	Canada	–	9,644,864	22.19
Breiner-8, 2016, [15]	2003	Canada	–	9,825,322	22.88
Breiner-9, 2016, [15]	2004	Canada	–	10,004,779	23.79
Breiner-10, 2016, [15]	2005	Canada	–	10,173,985	24.69
Breiner-11, 2016, [15]	2006	Canada	–	10,189,022	26.09
Breiner-12, 2016, [15]	2007	Canada	–	10,173,121	26.79
Breiner-13, 2016, [15]	2008	Canada	–	10,346,890	28.09
Breiner-14, 2016, [15]	2009	Canada	–	10,535,185	29.19
Breiner-15, 2016, [15]	2010	Canada	–	10,723,870	25.49
Breiner-16, 2016, [15]	2011	Canada	–	10,923,120	31.19
Breiner-17, 2016, [15]	2012	Canada	–	11,114,448	31.89
Breiner-18, 2016, [15]	2013	Canada	–	11,274,236	32.02
Maharaj, 2013, [87]	2013	USA	–	412,810	8.72
Gordon, 2015, [88]	2015	USA	–	265,844	77
Phillips-1, 1992, [89]	1980	USA	–	537,313	13.4
Phillips-2, 1992, [89]	1984	USA	–	556,338	14.2
Kurland, 1958, [90]	1954	USA	–	30,000	3.33
Alter, 1960, [91]	1956	USA	–	188,000	3.19
Kvirkveliia, 1986, [92]	1984	USA	–	555,851	14.2
Cisernos, 1996, [93]	1991	Cuba	–	5,782,309	2.92
Sanchez, 2002, [94]	2000	Colombia	–	5,300,000	2.77
Deffeminis, 1975, [95]	1975	Uruguay	–	2,700,000	6.29
Khedr, 2016, [96]	2016	Qena	–	9303	21.49
El-Tallawy, 2005, [97]	1997	Egypt	–	50,000	10
Gattellari, 2012, [98]	2012	Australia	–	21 874 920	2.49

### Systematic meta-analysis and descriptive review

**Fig. 2:**
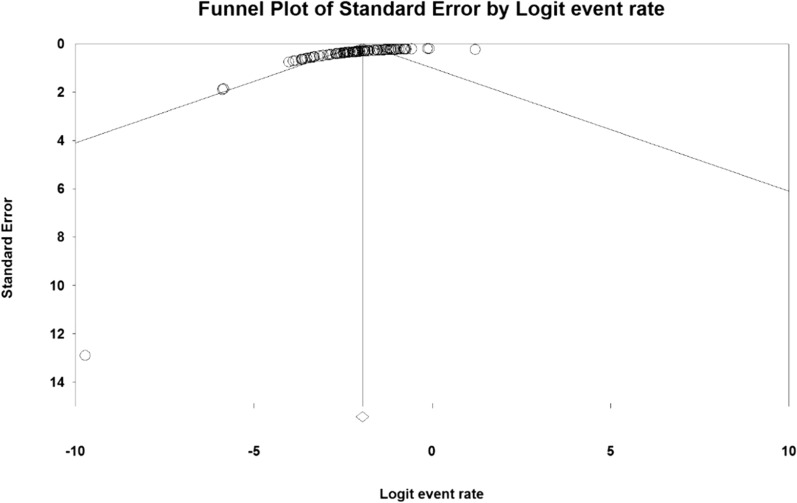
Funnel plot Results for estimating the prevalence of Myasthenia Gravis worldwide

**Fig. 3:**
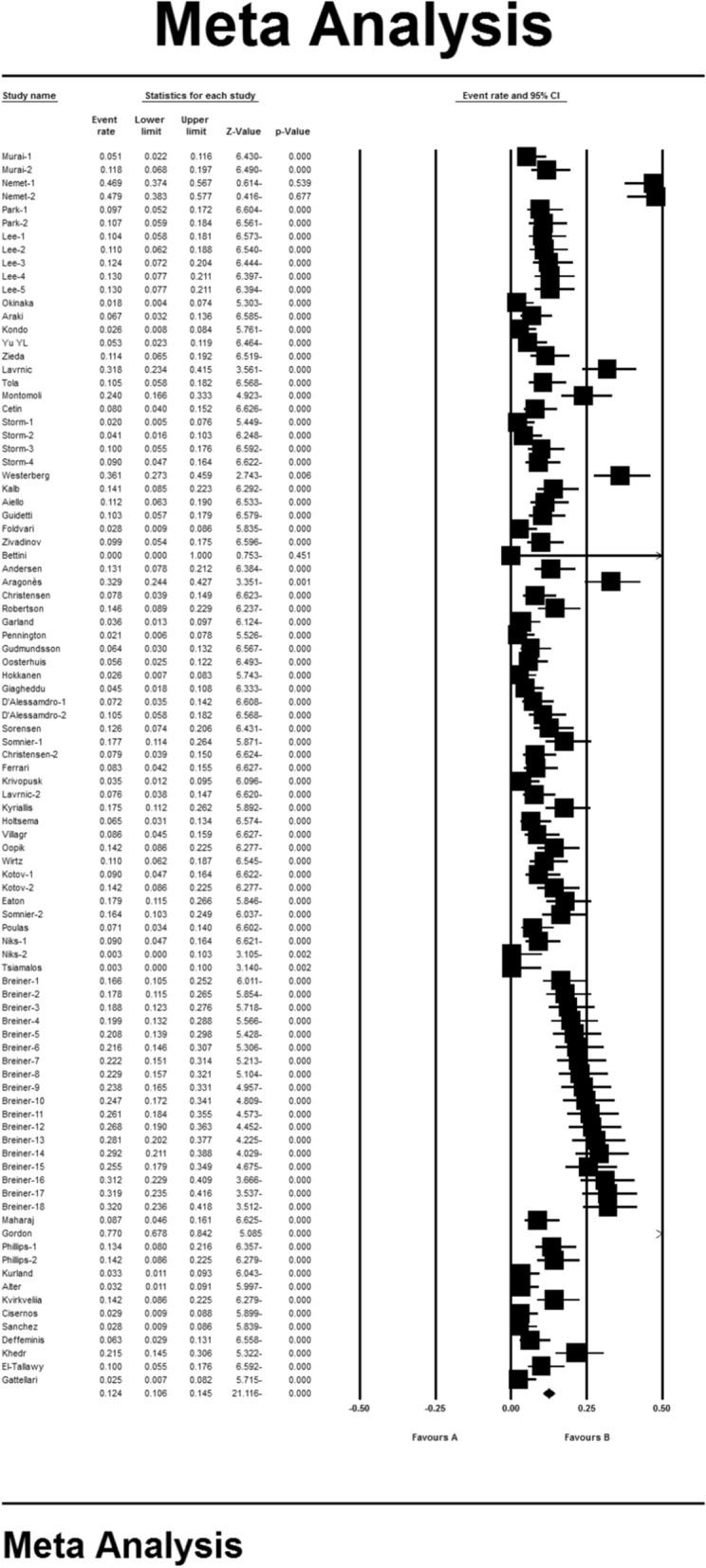
Estimation of the prevalence of Myasthenia Gravis in the world based on a random-effects model

The result of the I^2^ test for the prevalence of MG in the world indicates a significant heterogeneity between studies (I^2^ = 99.9), so the data were analyzed by meta-analysis using a random-effects model. Due to the high heterogeneity of the studies, sensitivity analysis was performed, and each study's effect on the final result and the degree of heterogeneity was evaluated. None (P = 0.103) (Fig. 2). As a result of the combination of studies, the overall estimate of the global prevalence of MG 1^2^. 4 people (95% confidence interval: 10-14-5.5) per 100,000 population was based on a random-effects model. The black square is the prevalence and the length of the line segment on which the 95% confidence interval per It is a study, the rhombus symbol shows the worldwide prevalence for all studies (Fig. 3). The highest prevalence was reported in Salvado et al. [61]; 3463 per 100,000 population and the lowest prevalence Bettini et al. [58]; 0.006 people reported per 100,000 population.

According to different reports of MG prevalence in different parts of the world, subgroup analysis by different continents (Asia, Europe, Africa and America) is reported in Table 2, which has the highest prevalence in the Americas with 19 people (95% CI 15–23.8) (Table 2).

**Table 2:** Prevalence of Myasthenia gravis by different continents

Continents	Number of articles	Sample size	I^2^	Begg and mazumdar rank correlation test	Prevalence per 100,000
Asia	15	603,327,498	91.8	0.101	10.9 (95% CI 6.4–17.9)
Europe	47	1,383,299,257	79.2	0.102	10 (95% CI 8.2–12.2)
America	28	195,218,499	87.9	0.110	19 (95% CI 15–23.8)
Africa	2	59,303	79.03	–	15.2 (95% CI 6.9–30.2)

### The analytical part of the study

**Fig. 4:**
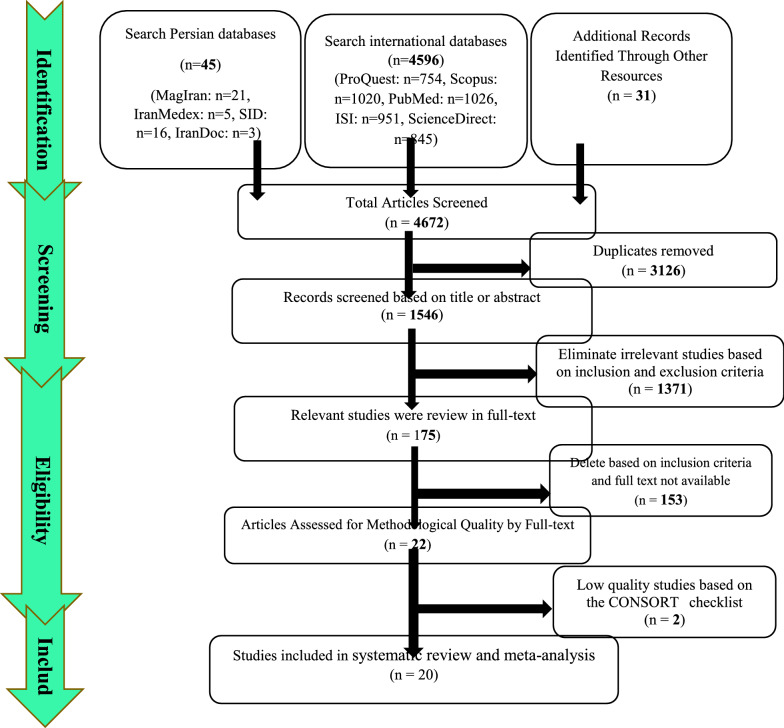
Preferred Reporting Items for Systematic Reviews and Meta-Analyses (PRISMA 2009) flow diagram Analytical section

Summary of how to enter articles: In the first stage, 4672 articles (4596 articles in international databases, 45 articles in Persian databases and 31 studies in reviewing the sources of articles) were found, and 3126 studies that were repeated in different databases were deleted. 1546 studies were entered in the screening stage, and based on the inclusion and exclusion criteria, the article was removed by reviewing the title and abstract of the 1992 studies. In the next stage (competency assessment), out of the remaining 175 studies from the screening stage, 183 articles were removed by reviewing the full text of the article because it was not relevant to the research. The remaining 22 articles were evaluated qualitatively by the CONSORT checklist, of which 2 studies were of low quality according to the criteria of this tool and were excluded from the study. Therefore, 20 articles related to the analytical part of the study were included in the systematic review and meta-analysis process (Fig. 4).

#### General characteristics of analytical studies

The total sample size was 643 in the drug group and 619 in the placebo group. The studies were published between 1976 and November 15, 2020. The smallest sample size was related to the study of Benatar et al. [109] with 6 patients in the drug group and 5 patients in the placebo group, and the largest sample size was related to the study of Sanders et al. [112] with 88 patients in the drug group and 88 patients in the placebo group. Summary of study characteristics including the name of the first author, year of publication, place of study, sample size, type of drug, and mean and standard deviation before and after the intervention of QMGS, Anti-AchR antibodies, SFEMG and MG-ADL indices are reported in Table 3.

**Table 3:** General characteristics of Analytical studies

Author, year, Reference	Country	Sample size		Variables	Mean ± SD drug group		Mean ± SD placebo group		Drug	Quality
		Placebo group	Drug group		Before	After	Before	After		
Immunoglobulin or plasma exchange										
Barth, 2011, [31]	Canada	41	–	QMGS	14.26 ± 4.0	10.96 ± 3.5	–	–	IVIG	High
Gajdos-1, 1997, [99]	France	46	–	Anti-AchR ab level, nmol/L	25 ± 65	17 ± 40	–	–	IVIG	Medium
Zinman, 2007, [100]	Canada	24	27	QMGS	12.3 ± 4.89	9.76 ± 4.0	12.5 ± 5.49	11.61 ± 4.5	IVIG	High
				SFEMG	85.2 ± 50.0	–	91.6 ± 37.9	–		
Wolfe, 2002, [101]	USA	6	9	QMGS	8.5 ± 1.8	8.5 ± 3.8	11.3 ± 5.6	9.7 ± 2.7	IVIG	High
				SFEMG	66.9 ± 21.0	56.6 ± 17.5	115 ± 84.2	94.1 ± 10.2		
				MG-ADL	5.3 ± 3.8	5.0 ± 2.0	6.0 ± 3.8	3.4 ± 2.4		
Gajdos-2, 2005, [30]	France	81	87	Anti-AchR ab level, nmol/L	12.0 ± 2.5	11.21 ± 14.02	10.0 ± 2.8	9.35 ± 15.07	IVIG	High
Gamez, 2019, [102]	Spain	25	22	QMGS	6.1 ± 3.8	5.2 ± 2.4	6.6 ± 3.5	7.1 ± 3.5	IVIG	High
				Anti-AchR ab level, nmol/L	14.4 ± 8.0	16.3 ± 7.4	11.3 ± 8.5	14.4 ± 8.5		
Barnett, 2013, [103]	USA	26	25	QMGS	12.2 ± 4.9	11.68 ± 4.0	12.4 ± 5.5	12.25 ± 3.5	IVIG	High
Zinman, 2008, [104]	Canada	26	26	QMGS	12.5 ± 5.5	11.5 ± 4.1	12.3 ± 4.9	14.0 ± 0.71	IVIG	High
Katzberg, 2014, [105]	Netherlands	24	27	QMGS	12.3 ± 3.5	10.22 ± 3.5	12.5 ± 1.9	12.09 ± 1.9	IVIG	High
				SFEMG	85.2 ± 3.0	76.3 ± 3.0	91.6 ± 4.1	86.3 ± 1.5		
Jacob, 2020, [106]	Japan	9	9	QMGS	18.1 ± 5.84	14.0 ± 3.6	18.2 ± 5.49	16.9 ± 2.8	Eculizumab	High
				MG-ADL	10.9 ± 3.37	5.6 ± 3.2	9.7 ± 2.4	7.6 ± 2.8		
Gajdos-3, 2008, [107]	France	87	85	QMGS	3.9 ± 1.1	1.36 ± 0.9	4.2 ± 0.70	3.31 ± 1.2	IVIG	High
Katzberg-2, 2012, [32]	Canada	24	81	QMGS	12.6 ± 3.6	12.4 ± 4.2	14.9 ± 3.2	14.4 ± 4.5	IVIG	Medium
				Anti-AchR ab level, μmol/L	–	166.2 ± 12.3	–	209.1 ± 11.6		
				SFEMG	86.6 ± 7.7	85.2 ± 6.5	122.5 ± 7.5	117.2 ± 6.3		
Howard, 2019, [108]	Netherlands	12	12	QMGS	11.8 ± 5.4	8.9 ± 3.8	14.5 ± 6.3	12.4 ± 4.6	Efgartigimod	High
				SFEMG	8.0 ± 2.2	3.6 ± 1.6	8.0 ± 3.0	5.9 ± 3.1		
Corticosteroids										
Benatar, 2016, [109]	USA	6	5	QMGS	6.0 ± 0.2	3.75 ± 0.9	6.5 ± 1.8	6.45 ± 1.3	Prednisone	High
Howard, 1976, [110]	USA	20	21	QMGS	8.18 ± 0.63	8.15 ± 0.32	7.97 ± 0.91	8.04 ± 0.47	Prednisone	Medium
Lindberg, 1998, [111]	USA	10	9	Anti-AchR ab level, μmol/L	354 ± 290	–	401 ± 264	–	Methylprednisolone	Medium
Mycophenolate										
Sanders, 2008, [112]	USA	88	88	QMGS	11.3 ± 1.2	7.3 ± 1.0	11.2 ± 1.3	7.9 ± 1.4	Mycophenolate	High
				MG-ADL	5.14 ± 0.77	2.71 ± 0.81	4.55 ± 0.79	2.74 ± 0.74		
Meriggioli, 2003, [21]	USA	7	7	QMGS	15.86 ± 15.2	13.0 ± 10.8	16.71 ± 12.3	16.43 ± 11.2	Mycophenolate	High
				Anti-AchR ab level, nmol/L	9.3 ± 2.3	3.32 ± 0.62	4.39 ± 0.9	3.81 ± 1.3		
				SFEMG	71.54 ± 11.3	60.52 ± 13.1	70.0 ± 13.2	69.26 ± 12.5		
Wolfe, 2008, [113]	USA	40	40	QMGS	12.9 ± 5.1	8.42 ± 5.1	–	–	Mycophenolate	Medium
				MG-ADL	6.9 ± 3.1	3.3 ± 3.3	7.2 ± 3.4	4.4 ± 3.7		
Group, 2008, [114]	USA	41	39	QMGS	13.3 ± 5.6	8.9 ± 5.1	12.5 ± 4.5	8.9 ± 5.0	Mycophenolate	High
				Anti-AchR ab level, nmol/L	13.6 ± 11.3	8.3 ± 9.5	23.0 ± 13.2	16.9 ± 10.8		
				MG-ADL	6.9 ± 3.1	3.3 ± 3.3	7.2 ± 3.4	4.4 ± 3.7		

### Immunoglobulin or plasma exchange drugs

A total of 13 studies examined the effect of immunoglobulin or plasma exchange drugs on MG patients. Studies were reported from 1997 to 2020. 11 studies examined the QMGS index, 4 studies the Anti-AChR antibodies, 4 studies the SFEMG index, and 3 the MG-ADL index.

### MG-ADL index

The Daily Living Activity Scale (MG-ADL) is an 8-item scale to assess secondary ocular disability (two items), bulbar (three items), respiratory (one item), limb (two items) related to myasthenia gravis effects. This scale has a linear rating from zero to 3, and its overall scoring range is from zero to 24 [115]. The patient's MG-ADL questionnaire is completely reported without training and specialized equipment and usually lasts less than five minutes [116].

Based on the present meta-analysis results between studies, there is a lot of heterogeneity (I^2^ = 78.5), so the stochastic effects model was used to combine the studies and the final result. Begg and Mazumdar rank correlation to test the publication bias in the studies for the MG-ADL index (P = 1.000) (Table 4).

**Table 4:** Mean and standard deviation as well as the difference between the mean of drug evaluation indices before and after the intervention in the studied groups

Drugs	Index	I^2^	Begg and mazumdar rank correlation test	Mean ± SD before intervention (drug group)	Mean ± SD after intervention (drug group)	Mean difference and standard deviation before and after the intervention
Mycophenolate	MG-ADL	85.3	1.000	5.9 ± 0.87	7.5 ± 4.09	1.4 ± 0.9
	SFEMG	0	–	71.5 ± 11.3	60.5 ± 13.1	0.9 ± 0.56
	Anti-AChR antibodies	79.1	–	11.1 ± 2.1	5.5 ± 2.4	1.9 ± 1.5
	QMGS	67.9	0.734	12.3 ± 0.71	8.1 ± 0.59	1.4 ± 0.77
Immunoglobulin or plasma exchange	MG-ADL	78.5	1.000	8.2 ± 1.3	4.4 ± 0.84	1.3 ± 0.63
	SFEMG	43.5	1.000	82.2 ± 1.43	54.5 ± 27.5	1.5 ± 0.73
	Anti-AChR antibodies	99.8	1.000	10.8 ± 4.6	52.7 ± 34.1	–2.006 ± 0.78
	QMGS	98.6	0.391	11.2 ± 1.6	9.4 ± 1.8	0.62 ± 0.28
Corticosteroids	QMGS	99.4	–	7.08 ± 1.09	5.9 ± 2.2	1.64 ± 1.6

**Fig. 5:**
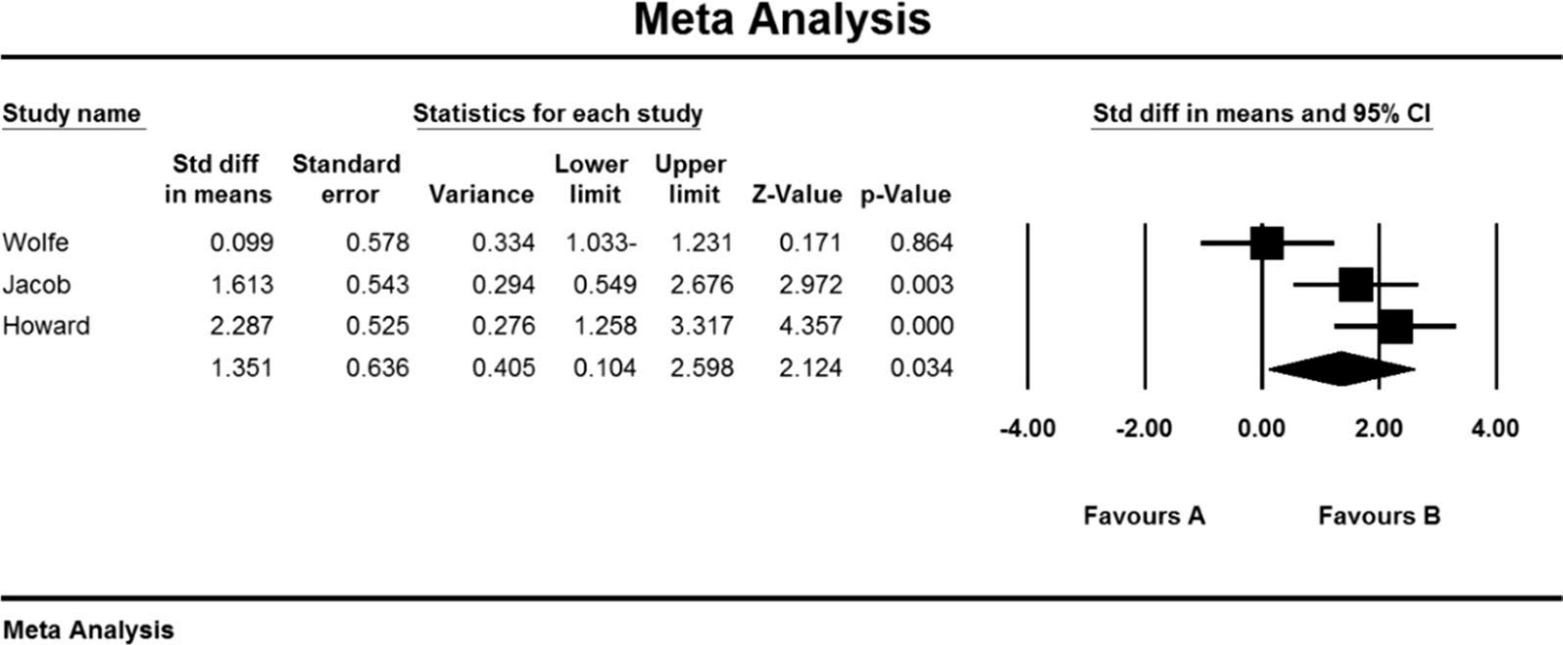
Accumulation chart of studies included in the meta-analysis based on the difference between the mean MG-ADL index before and after the intervention for Immunoglobulin or plasma exchange

As a result of the combination of studies, the mean score of MG-ADL indices before the intervention in the drug group was 8.2 ± 1.3. After the intervention was 4.0 ± 4.84 (Table 4), as well as the difference between the mean of the MG-ADL index before and after the intervention, 1.3 ± 0.63 was obtained (P  < 0.01) (Fig. 5), which indicates the positive effect of Immunoglobulin or plasma exchange on the reduction of MG-ADL index.

### SFEMG index

Single-fiber electromyography (SFEMG) is an efficient tool to investigate neurotransmitter disorders. In this method, with the help of bipolar needle electrodes, the action potential of two adjacent muscle fibres belonging to a motor unit that have been activated voluntarily or electrically stimulated can be recorded [117]. This technique is more time consuming than conventional EMG, and patient cooperation in this method is effective because even small movements by the patient can lead to loss or change of action potential [118].

**Fig. 6:**
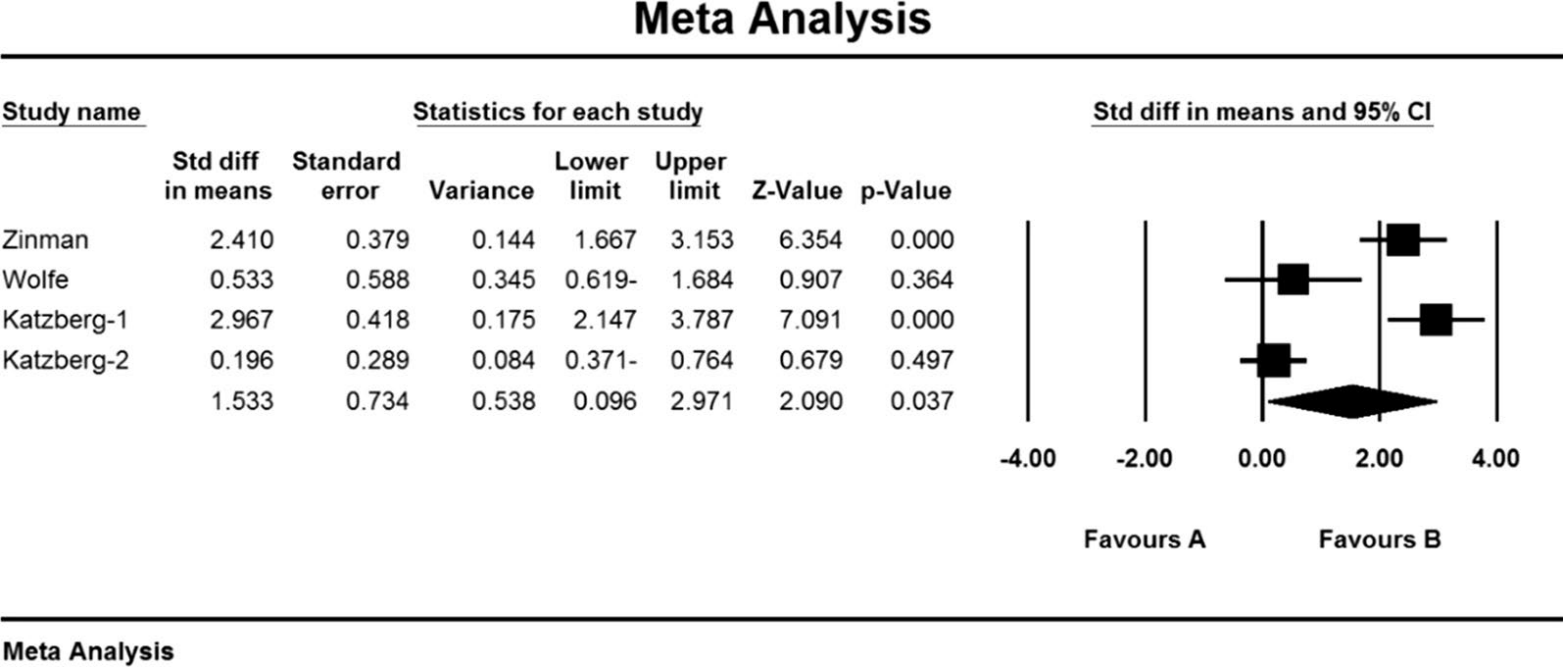
Accumulation chart of studies included in the meta-analysis based on the difference between the mean SFEMG index before and after the intervention for Immunoglobulin or plasma exchange

Based on the present meta-analysis results between studies, there is a lot of heterogeneity (I^2^ = 43.5), so the stochastic effects model was used to combine the studies and the final result. Begg and Mazumdar rank correlation tests were not available in studies for the SFEMG index (P = 1.000) (Table 4). As a result of the combination of studies, the mean score of SFEMG indices before the intervention in the drug group was 82.2 ± 1.43, and after the intervention was 54/5 ± 27/5 (Table 4), also, the difference between the mean of SFEMG index before and after the intervention was 1.5 ± 0.73 (P < 0.01) (Fig. 6), which indicates the positive effect of immunoglobulin or plasma exchange on the reduction of SFEMG index.

### Anti-AChR antibodies index

Weakness and fatigue in myasthenia gravis are caused by a decrease in acetylcholine receptors due to an autoimmune attack of antibodies at the neuromuscular junction. Specific antibodies induce this autoimmune response against the acetylcholine receptor by blocking or binding to the receptor or postsynaptic membrane damage [119].

**Fig. 7:**
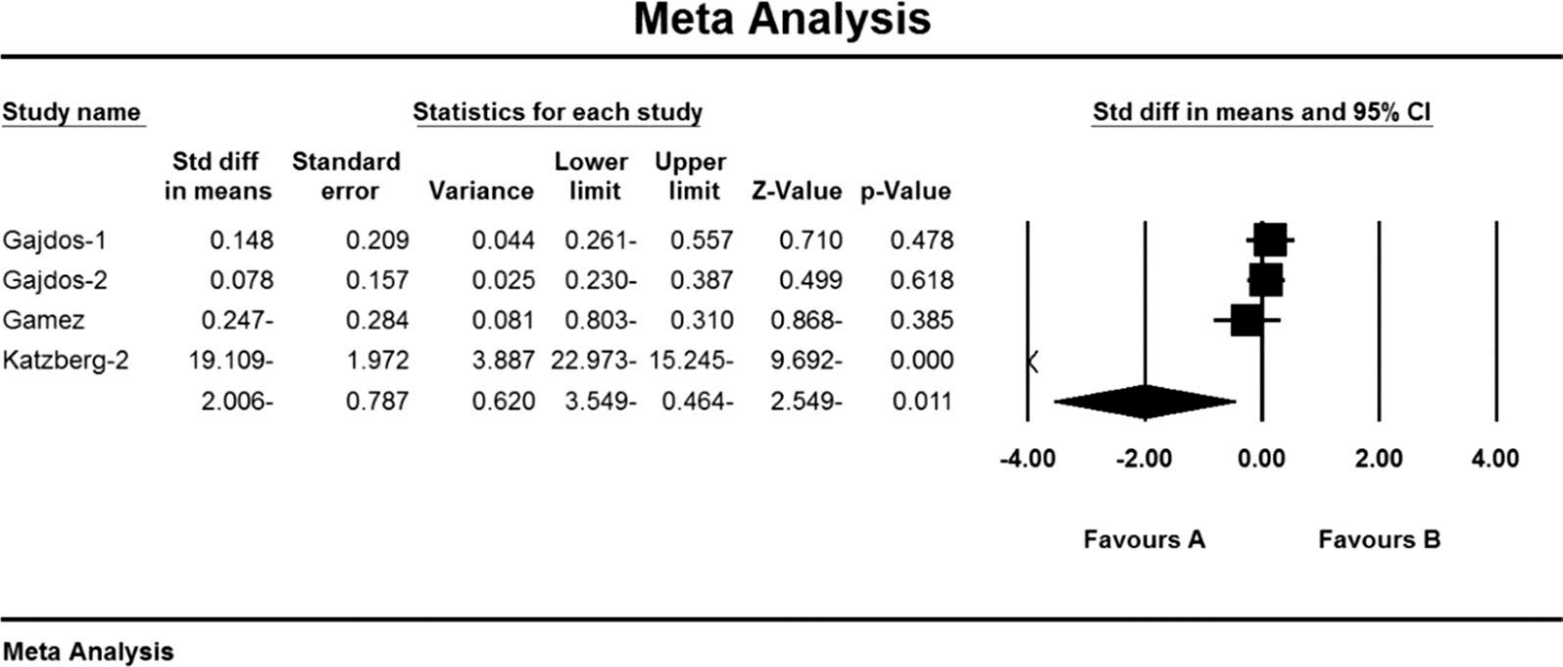
Accumulation chart of studies included in the meta-analysis based on the mean difference of the anti-AChR antibodies index before and after the intervention for Immunoglobulin or plasma exchange

Based on the present meta-analysis results, there is a lot of heterogeneity between studies (I^2^ = 99.8), so the stochastic effects model was used to combine the studies and the final result. Begg and Mazumdar rank correlation test Emission bias was not presented in studies for Anti-AChR antibodies index (P = 1.000) (Table 4). As a result of the combination of studies, the mean score of Anti-AChR antibodies before the intervention in the drug group was 10.8 ± 4.6 and after the intervention was 52.7 ± 34.1 (Table 4). AChR antibodies were obtained before and after the intervention at − 2.006 ± 78.7 (P < 0.01) (Fig. 7), indicating that Immunoglobulin or plasma exchange did not affect the Anti-AChR antibodies index. QMGS index.

### QMGS index

Myasthenia Gravis Quantitative Score (QMGS) is a 13-item scale developed by Tindall et al. [120] and modified by Barohn et al. [121] to be used to determine the severity of myasthenia gravis. This scale measures ocular, bulbar, respiratory, and limb function and scores each finding from zero (no myasthenic findings) to 39 (maximum myasthenic defects) [122, 123].

Based on the present meta-analysis results, there is a lot of heterogeneity between studies (I^2^ = 98.6), so the random-effects model was used to combine the studies and the final result. Begg and Mazumdar rank correlation test was not possible in the studies for the QMGS index (P = 0.391) (Table 4).

**Fig. 8:**
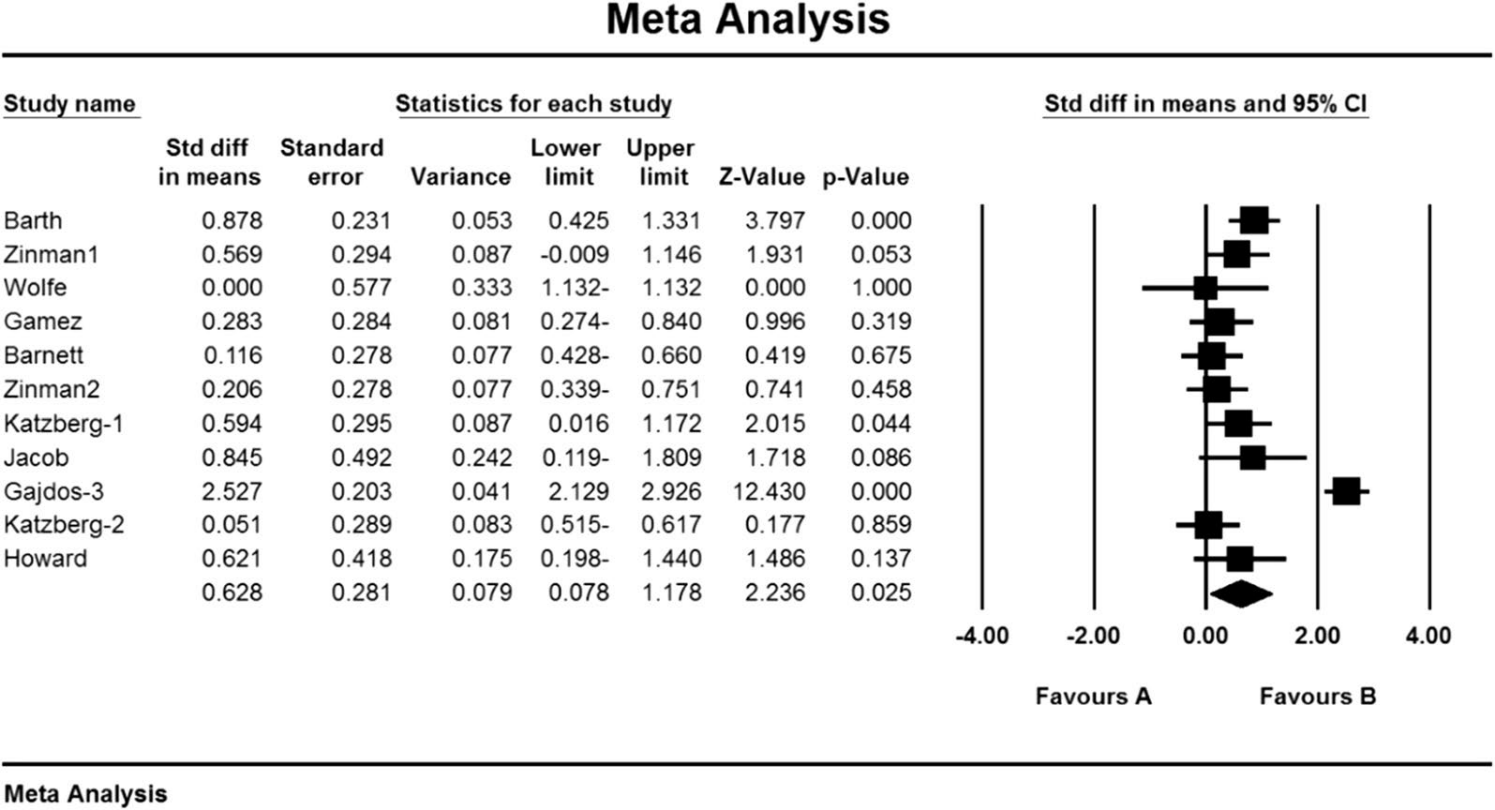
Accumulation diagram of meta-analysis studies based on mean differences in mean QMGS before and after intervention for Immunoglobulin or plasma exchange

As a result of the combination of studies, the mean score of QMGS indices before the intervention in the drug group was 11.2 ± 1.6 and after the intervention was 9/1 ± 4/8 (Table 4), as well as the difference between the mean of the QMGS index before and after the intervention. 0.62 ± 0.28 was obtained (P <  0.01) (Fig. 8), which indicates the positive effect of Immunoglobulin or plasma exchange on QMGS index reduction.

### Mycophenolate

In total, 4 studies examined the effect of Mycophenolate on MG patients. 4 studies reviewed the QMGS index, 2 studies the Anti-AChR antibodies index, 1 study the SFEMG index and 3 studies the MG-ADL index.

### MG-ADL index

Based on the results of the present meta-analysis studies, there is a lot of heterogeneity (I^2^ = 85.3), so the stochastic effects model was used to combine the studies and the outcome. Begg and Mazumdar rank correlation test of publication bias was not possible in the studies for MG-ADL index (P = 1.000) (Table 4).

**Fig. 9:**
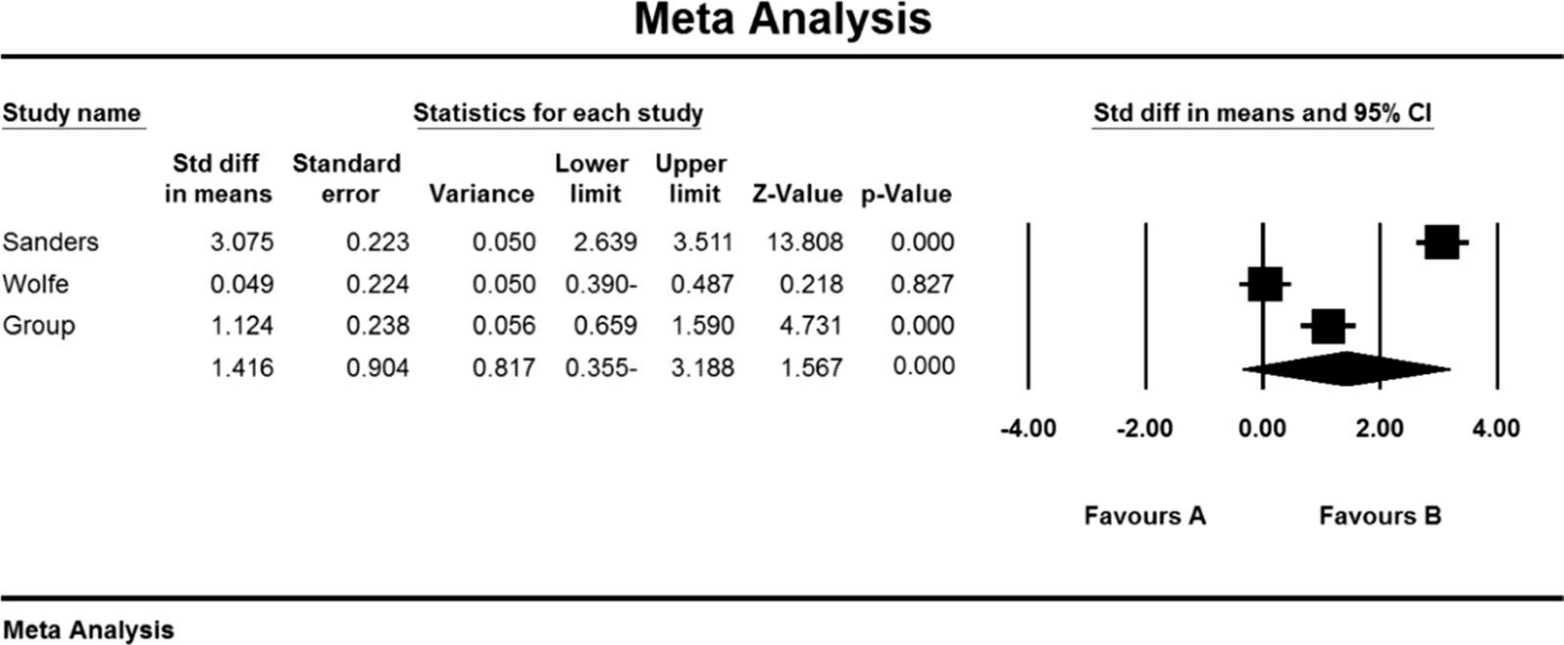
Accumulation chart of studies entered for meta-analysis based on the difference between the mean MG-ADL index before and after intervention for Mycophenolate

As a result of the combination of studies, the mean score of MG-ADL indices before the intervention in the drug group was 5.9 ± 0.87 and after the intervention was 7.4 ± 5.09 (Table 4), as well as the difference between the mean of the MGADL index before and after The intervention showed 1.4 ± 0.9 (P < 0.01) (Fig. 9) which indicates the positive effect of Mycophenolate on the reduction of MG-ADL index.

### SFEMG index

Based on the present meta-analysis results between studies, according to a study, there was no heterogeneity (I^2^ = 0), so the fixed effects model was used to combine the study and the final result. It was not possible to perform Begg and Mazumdar rank correlation test in the studies for the SFEMG index according to the study of only one research (Table 4).

As a result of the combination of studies, the mean score of SFEMG indices before the intervention in the drug group was 71.5 ± 11.3 and after the intervention was 60.5 ± 13.1 (Table 4), as well as the difference between the mean of the SFEMG index before and after the intervention. 0.9 ± 0.56 was obtained (P < 0.01), indicating Mycophenolate's positive effect on SFEMG index reduction.

### Index of anti-AChR antibodies

Based on the present meta-analysis results between studies, there is a lot of heterogeneity (I^2^ = 79.1), so the stochastic effects model was used to combine the studies and the final result. It was not possible to perform Begg and Mazumdar rank correlation test, publication bias in studies for Anti AChR antibodies index due to review of only 2 studies (Table 4).

**Fig. 10:**
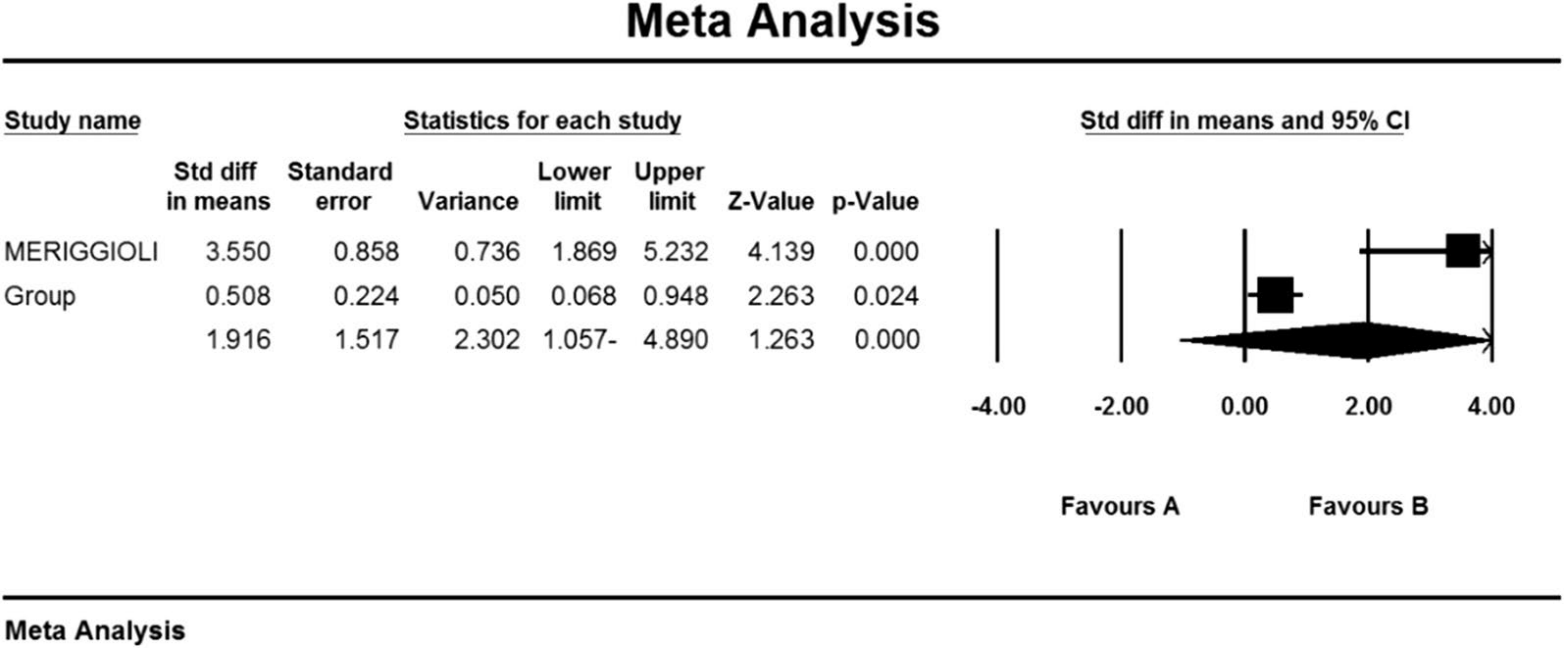
Accumulation chart of studies included in the meta-analysis based on the mean difference of the anti-AChR antibodies index before and after the intervention for Mycophenolate

As a result of the combination of studies, the mean score of Anti-AChR antibodies before the intervention in the drug group was 11.1 ± 2.1 and after the intervention was 5.5 ± 2.4 (Table 4) and the difference between the mean of the anti-AChR index. Antibodies were obtained before and after the intervention (1.9 ± 1.5 (P < 0.01) (Fig. 10)), which indicates the positive effect of Mycophenolate on the Anti-AChR antibodies index.

### QMGS index

Based on the present meta-analysis results between studies, there is a lot of heterogeneity (I^2^ = 67.9), so the stochastic effects model was used to combine the studies and the final results.

**Fig. 11:**
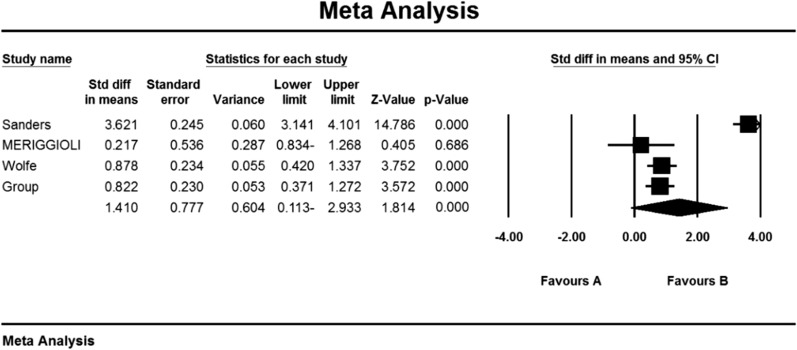
Accumulation chart of studies entered for meta-analysis based on the mean difference of QMGS index before and after intervention for Mycophenolate

As a result of the combination of studies, the mean score of QMGS indices before the intervention in the drug group was 12.3 ± 0.71 and after the intervention was 8.0 ± 0.59 (Table 4). It was obtained 1.4 ± 0.77 (P  < 0.01) (Fig. 11), indicating Mycophenolate's positive effect on QMGS index reduction.

### Corticosteroids

In the study of studies in the field of corticosteroids, only the QMGS index could be examined. Based on this, 3 studies examined the effect of corticosteroids on MG patients. In the study of Benatar et al. [109], the QMGS index before the intervention in the placebo group was 6.5 ± 1.8 units and in the drug group was 6.0 ± 0.2 units, and after the intervention in the placebo group decreased by 0.05 units (P   > 0.05). There was a significant decrease of 2.25 units (P < 0.05) [109]. Also, in the study of Howard et al. [110] QMGS index before the intervention in the placebo group was 7.97 ± 0.91 units and in the drug group was 8.18 ± 0.63 units and after the intervention in the placebo group increased by 0.07 units (P    > 0.05) and in the drug group had a decrease of 0.03 units (P  > 0.05) [110]. In the study of Lindberg et al. [111], The anti-AChR antibodies index was reported before intervention in the placebo group of 264 ± 401 (μmol/L) and in the drug group of 354 m 290 (μmol/L) [111].

### QMGS index

Based on the present meta-analysis results between studies, there is a lot of heterogeneity (I^2^ = 99.4), so the stochastic effects model was used to combine the studies and the final result of the outcomes. According to the review of only two studies, it was not possible to use the Begg and Mazumdar rank correlation test for the QMGS index studies according to the review of only 2 studies (Table 4).

**Fig. 12:**
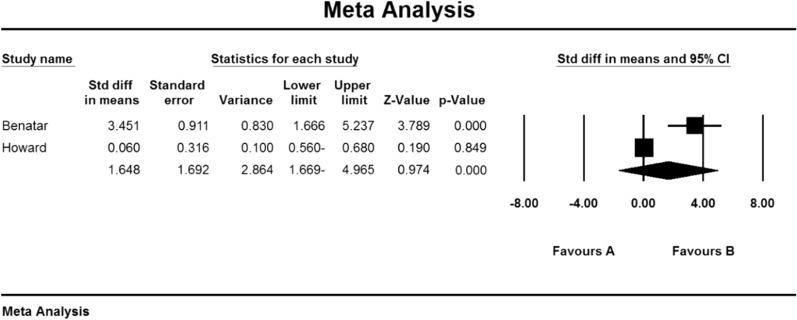
Accumulation diagram of studies included in the meta-analysis based on the difference between the mean QMGS index before and after the intervention for Corticosteroids

As a result of the combination of studies, the mean score of QMGS indices before the intervention in the drug group was 7.08 09 1.09 and after the intervention was 5.9 2 2.2 (Table 4), as well as the difference between the mean scores of the QMGS index before and after the intervention. 1.64 1 1.6 was obtained (P.010.01) (Fig. 12), which indicates the positive effect of corticosteroids on reducing the QMGS index.

## Discussion

Myasthenia gravis (MG) is the largest group of neuromuscular disorders caused by autoimmune antibodies against postsynaptic components of the voluntary muscle endplate [124–126]. Acetylcholine receptor antibodies (AChR), muscle-specific kinase (MuSK), and lipoprotein-associated protein (LRP4) have been well established as sensitive diagnostic markers and pathogens, in addition to antibodies in the classification of patients with Myasthenia gravis also play a key role [127].

Although the clinical features of MG can vary, increasing muscle weakness with continued skeletal muscle activity is one way to diagnose the disease [128]. Unlike ocular involvement, which is often asymmetric and involves several muscles, the pattern of muscle involvement in myasthenia gravis is usually symmetrical. Muscle weakness usually increases with exercise and frequent muscle use, and its intensity varies from day to day and fluctuates throughout the day [129].

In the current systematic review and meta-analysis study, the overall prevalence of MG in the world; 12.4 people per 100,000 population were obtained. Most prevalent in Salvado et al. [61]; 3463 per 100,000 population and the lowest prevalence Bettini et al. [58]; 0.006 people per 100,000 population reported.

Due to the different reports of MG prevalence in different parts of the world, a detailed study of the prevalence of this disease in different continents in order to pay more attention to planners and its consequences seemed necessary. Therefore, according to the subgroup analysis by different continents (Asia, Europe, Africa, and America), the highest prevalence of myasthenigraphy was reported in the Americas with 19 per 100,000 people and the lowest in continental Europe with 10 per 100,000 people.

Symptomatic, safe, and supportive approaches are very effective in treating myasthenia gravis, and treatment should be aimed at complete or almost complete drug recovery [130]. Most patients with myasthenia gravis to achieve therapeutic goals of full physical function or relatively high quality of life need immunosuppressive drugs. Immunosuppressive drugs are prescribed to all patients who respond only to symptomatic and supportive treatment [131].

Only the QMGS index could be assessed in studies of corticosteroids, which measures the severity of myasthenia gravis in 13 items [120]. The mean score of the QMGS index before and after the intervention in the drug group was 7.08 09 1.09 and 5.9 2 2.2, which indicates the positive effect of corticosteroid use on reducing the QMGS index improving myasthenia gravis.

Oral corticosteroid therapy has been used since the 1950s with a dramatic improvement in approximately 70 to 80% of patients with myasthenia gravis [132, 133]. The usefulness of oral steroids is determined by the occurrence of a wide range of dose and time-dependent side effects [134, 135]. Intermittent intravenous methylprednisolone (IVMP) is used to treat several autoimmune disorders, including MG, on the assumption that it is more effective and has fewer side effects than oral steroids [136]. IVMP is also effective in severe cases of MG [137].

Mycophenolate mofetil (MMF) is an immunosuppressive agent that is primarily used to prevent acute rejection of organ transplants [138] which have reported preliminary use of this drug in the treatment of myasthenia gravis [139].

Regarding the effectiveness of mycophenolate mofetil, the mean score of MG-ADL index before and after the intervention in the drug group was 5.9 87 0.87 and 7.4 09 5.09, respectively. This scale assesses daily life activity in people with myasthenia gravis through 8 items [115]. The mean score of the SFEMG (single-strand electromyography) index, which is used to evaluate neuromuscular site abnormalities [117], was reported to be 71.5 ± 11.3 and 60.13 5 5.1, respectively, before and after the intervention in the drug group. Also, the mean score of Anti-AChR antibodies before and after the intervention in the drug group was 5/5 ± 2/4 and 11/2 ± 1/1. The mean score of QMGS indices before and after the intervention in the drug group was 12.3 ± 0.71 and 8.0 ± 1.59, which the results show the positive effect of using Mycophenolate on reducing the above 4 indicators and thus improving the treatment status of patients with MG.

Certain cure requires suppression or modulation of the immune system by intravenous immunoglobulin (IVIg) or plasma replacement (PLEX) [140]. Immune system modification is used when rapid recovery is required, such as exacerbated myasthenia gravis, power optimization before thymectomy, and patients who do not tolerate and respond adequately to immunosuppressive drugs [100, 141, 142]. In recent years, the administration of 2 g/kg intravenous IVIg immunoglobulin has been proven to treat moderate to severe myasthenia gravis and is continuously used to manage intensified MG [143].

Therapeutic plasmapheresis or plasma replacement (PLEX) is the first line of treatment in patients with myasthenia gravis with respiratory failure, inability to swallow, myasthenic crisis, or inadequate response to drug therapy [144, 145]. In therapeutic plasmapheresis, plasma containing pathogenic antibodies is separated from the patient's blood and returned to other cells. Plasma replacement is prescribed five times in 10 to 14 days, through which and by repeating it, plasma levels of acetylcholine receptor antibody are reduced, and clinical improvement is achieved [119].

Due to the high prevalence of myasthenia gravis globally and its many negative consequences for individuals and society. Therefore, it seems useful to take measures to achieve better therapies or to use supportive therapies to reduce the symptoms of the disease. Common drug treatments in MG were evaluated to show the effectiveness of immunosuppressive drugs, including steroids and their modulators, including intravenous immunoglobulin (IVIg and plasma replacement) (PLEX). These studies can provide useful information to health care providers, enrich health care interventions, improve the quality of services, and ultimately improve the quality of life of these people. Therefore, it is suggested that physicians and the health care system give these drug classes more attention.

The application of nanotechnology is promising, given frustrating problems in therapeutic neurology [146]. Nanotechnology involves the manipulation of technological machinery at the atomic scale. For perspective, a nucleus is about 6 μm across, a ribosome 20 nm in diameter, and a single strand of DNA 2 nm wide [146]. A typical human being is composed of 100 trillion cells. Nanotechnology has created novel devices for the treatment of various neurological diseases. Shrinkage of machinery, chip-based technologies, and the creation of unprecedented nanomaterials are contributing immensely to the reduction of morbidity [146, 147]

Considerable efforts are being focused on using nanoneuromedicine for disease treatment in the research laboratory. In the case of neurodegenerative diseases such as myasthenia gravis (MG), Alzheimer's disease (AD), Parkinson's disease (PD), amyotrophic lateral sclerosis (ALS), and multiple sclerosis (MS), nanomedicines have emerged as promising treatment options. Pathophysiological processes involving neuron inflammation and protein misfolding initiate a degeneration cycle within the cell. This can be thwarted using better drug targeting. Diagnosing and monitoring the end-effects of therapeutics is possible using nanoneurotechnology [146–148].

In addition to what has been said, the interest in graphene-based nanomaterials (GBNs) application in nanomedicine, particularly neurology, steadily increased in the last decades. GBNs peculiar physical–chemical properties allow the design of innovative therapeutic tools to manipulate biological structures with subcellular resolution [148, 149]. Based on the study, it can also be said that to develop effective antioxidant therapies the best strategy may be to create new nanoscale drug delivery systems [150].

## Limitations

Among the limitations of this study, it can be asserted that some samples were not based on random selection. Also, non-uniform reporting of articles, inconsistent implementation method, non-copying and unavailability of the full text of articles presented at the conference can be mentioned as other limitations.

## Suggestion for future works

The meta-analysis results enable the overall prevalence to be presented to the policy-maker and thus manage the cost, time and future diagnostic and treatment decisions commensurate with the overall prevalence. A systematic review also reveals drugs effective in treating myasthenia gravis, which can guide physicians and encourage the researcher to conduct future clinical trial studies and a network meta-analysis to determine therapeutic supplements for the disease.

## Conclusion

The results of systematic review of drug evaluation in patients with myasthenia gravis showed that Mycophenolate and Immunoglobulin or plasma exchange drugs have positive effects in the treatment of MG. It also represents the positive effect of immunoglobulin or plasma exchange on reducing SFEMG index and QMGS index and the positive effect of Mycophenolate on reducing MG-ADL index, SFEMG and Anti-AChR antibodies index. In addition to what was mentioned, based on a meta-analysis of the random-effect model, the overall prevalence of MG in the world is 12.4 people per 100,000 populations, which indicates the urgent need for the attention of officials and specialists to this disease for prevention and treatment.

## Data Availability

Datasets are available through the corresponding author upon reasonable request.

## Abbreviations

SID: Scientific Information Database
MESH: Medical Subject Headings
WoS: Web of science
PRISMA: Preferred Reporting Items for Systematic Reviews and Meta-Analysis
CONSORT: Consolidated Standards of Reporting Trials
STROBE: Strengthening the reporting of observational studies in epidemiology for a cross-sectional study
MG: Myasthenia Gravis
Anti-AChR ab: Anti-acetylcholine receptor antibodies
IVIG: Intravenous immunoglobulin
QMGS: Quantitative Myasthenia Gravis Score
MG-ADL: Myasthenia Gravis Activities of Daily Living
SFEMG: Single-fibre electromyography
